# Atypical Acute Motor Axonal Neuropathy (AMAN) Presenting Concurrently With Community-Acquired Pneumonia and Respiratory Failure: A Case Report

**DOI:** 10.7759/cureus.97759

**Published:** 2025-11-25

**Authors:** Mohit Vaid, Monika Singh

**Affiliations:** 1 Internal Medicine, Vardhman Mahavir Medical College (VMMC) and Safdarjung Hospital, New Delhi, IND

**Keywords:** acute motor axonal neuropathy (aman), atypical gbs presentations, community-acquired pneumonia (cap), flaccid quadriparesis, guillain-barré syndrome (gbs)

## Abstract

A 36-year-old female presented with rapidly ascending, flaccid quadriparesis, bulbar, and respiratory failure requiring four days of mechanical ventilation. This presentation was highly atypical, as the neurological symptoms commenced virtually concurrently with an active community-acquired pneumonia (CAP), noted on the fourth day of her febrile prodrome, departing from the classic post-infectious timeline of Guillain-Barré syndrome (GBS). Initial investigations ruled out myopathy (normal creatine phosphokinase (CPK)/creatine kinase-MB (CK-MB)), while subsequent studies established the diagnosis. Nerve conduction velocity (NCV) confirmed non-length-dependent axonal motor polyneuropathy, and cerebrospinal fluid analysis showed albuminocytologic dissociation. The prompt suspicion and diagnosis of acute motor axonal neuropathy (AMAN) allowed for initiation of intravenous immunoglobulin (IVIG) and broad-spectrum antibiotics for the CAP, which resulted in a successful clinical course, including successful extubation, and impressive motor recovery upon discharge. This case report highlights the critical importance of recognizing parainfectious AMAN presentations to ensure timely immunomodulatory therapy in the setting of severe concurrent systemic illness.

## Introduction

Guillain-Barré syndrome (GBS) stands as the world's most frequent etiology of acute, flaccid paralysis, affecting approximately one to two individuals per 100,000 annually [1]. This is a highly heterogeneous collection of acute, immune-mediated polyneuropathies, almost invariably following a respiratory or gastrointestinal infection [1]. This preceding infection initiates an autoimmune cascade, which happens through molecular mimicry, leading to the immune system attacking components of the peripheral nervous system. The disorder primarily includes two major subtypes: acute inflammatory demyelinating polyneuropathy (AIDP) and acute motor axonal neuropathy (AMAN) [2]. AMAN is more prevalent across Asian demographics; it specifically damages the motor axons and nodes of Ranvier (small gaps in the myelin sheath), resulting in severe motor deficits with minimal or no corresponding sensory symptoms [3]. Pathophysiologically, AMAN is driven by acute Wallerian-like degeneration (a process where the part of the axon distal to the injury rapidly breaks down) of the motor axons, often mediated by autoantibodies (such as anti-GM1 or anti-GD1a) triggered by infectious agents like *Campylobacter jejuni* [2].

The classical presentation of GBS involves symmetrical, ascending weakness that evolves over a maximum of four weeks, beginning days to weeks after the initial prodromal infection has subsided. Crucially, the occurrence of GBS that develops in a parainfectious manner, coinciding directly with the acme of a severe, ongoing systemic infection, such as florid pneumonia, is exceedingly rare and poses a critical diagnostic difficulty [4]. This deviation from the expected post-infectious timeline obscures the diagnosis, immediately widening the differential to include critical illness polyneuropathy, infectious myelitis, and other conditions commonly seen in acutely ill patients. Failing to promptly recognize this rapid-onset parainfectious AMAN, especially when coupled with the respiratory compromise already imposed by the concurrent pneumonia, dramatically elevates the risk of adverse outcomes. Therefore, recognizing this unusual presentation is crucial. This case report aims to detail a case of rapidly progressive AMAN presenting in this highly atypical parainfectious context, beginning with the fourth day of severe community-acquired pneumonia (CAP), thus underscoring the imperative for immediate diagnosis and aggressive treatment.

## Case presentation

A previously healthy 36-year-old female presented to the emergency department on the second day of neurological weakness with acute, rapidly progressing muscle weakness. Her illness began with a constitutional syndrome characterized by a low-grade fever (maximum temperature 100.4), a productive cough with yellowish-green expectoration, and generalized malaise four days prior to admission.

The neurological symptoms began insidiously two days prior to admission (corresponding to Day 3 of the fever prodrome) with difficulty climbing stairs and getting up from a squatting position, indicative of proximal lower limb weakness. The progression was dramatic: within 24 hours, the weakness had ascended to involve the upper limbs. By the time of presentation (Day 2 of weakness), the patient exhibited profound, flaccid quadriparesis.

On examination, she was conscious but distressed, with signs of respiratory distress (shallow breathing, accessory muscle use present). A neurological examination revealed symmetrical motor weakness, graded 2/5 in all four limbs (as per Medical Research Council grading), with a complete absence of deep tendon reflexes. Sensory examination was entirely normal. Crucially, bulbar involvement was evident, presenting as severe dysphonia and mild nasal regurgitation, with impending airway compromise. Given the rapid progression with involvement of the respiratory and bulbar musculature, she was immediately transferred to the intensive care unit. Within two hours of arrival, she developed severe hypoventilation and was emergently intubated and placed on mechanical ventilation for airway protection and respiratory support.

**Figure 1:**
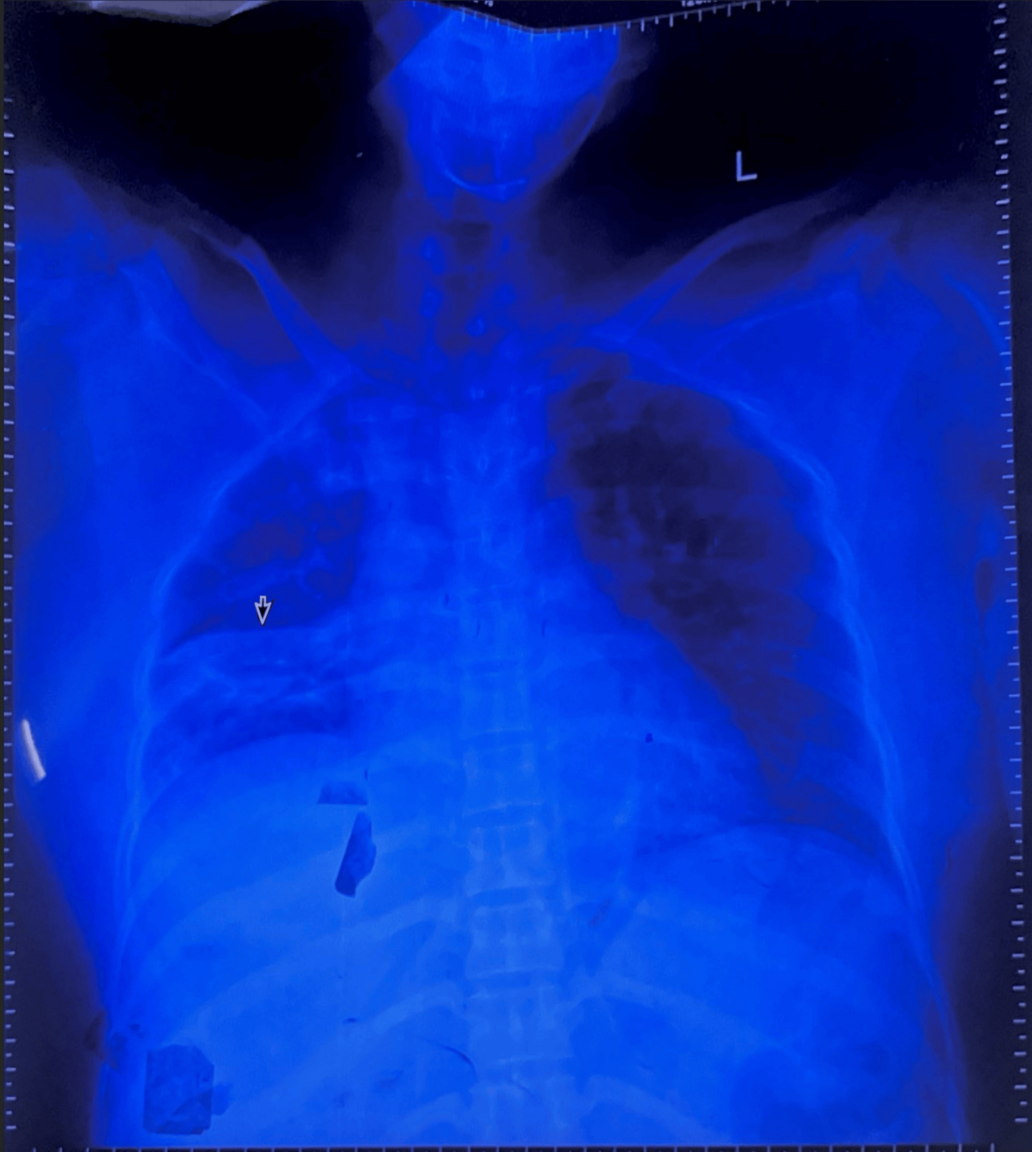
Chest X-ray revealed opacities consistent with consolidation in the right middle zone and right lower zone Arrow: consolidation in the right middle zone and right lower zone.

Initial laboratory and specialized investigations performed are summarized in Table 1, and the detailed nerve conduction velocity (NCV) and CSF findings were central to the diagnosis. The chest X-ray revealed opacities consistent with consolidation in the right middle zone and right lower zone (Figure 1), confirming an active CAP.

**Table 1:** Initial laboratory and specialized investigations performed NCV: nerve conduction velocity; CMAP: compound muscle action potential; SNAP: sensory nerve action potential

Investigation	Result	Unit	Significance	Relative Day Performed
Creatine Phosphokinase (CPK)	107.8	U/L	Normal (rules out significant myopathy/rhabdomyolysis)	Day 2 of Weakness
Creatine Kinase-MB (CK-MB)	21.9	U/L	Normal	Day 2 of Weakness
Procalcitonin (PCT)	0.13	ng/ml	Low likelihood of severe systemic bacterial infection	Day 3 of Weakness
Chest X-Ray	Right Middle + Lower Zone Consolidation		Confirms community-acquired pneumonia	Day 2 of Weakness
NCV Findings	Reduced CMAP, Preserved SNAP		Pure motor, non-length-dependent axonal polyneuropathy	Day 5 of Weakness
CSF Protein	150	mg/dL	Elevated	Day 9 of Weakness
CSF Total Leukocyte Count (TLC)	2	cells/uL	Normal (confirms albuminocytologic dissociation)	Day 9 of Weakness

Management and outcome

**Figure 2:**
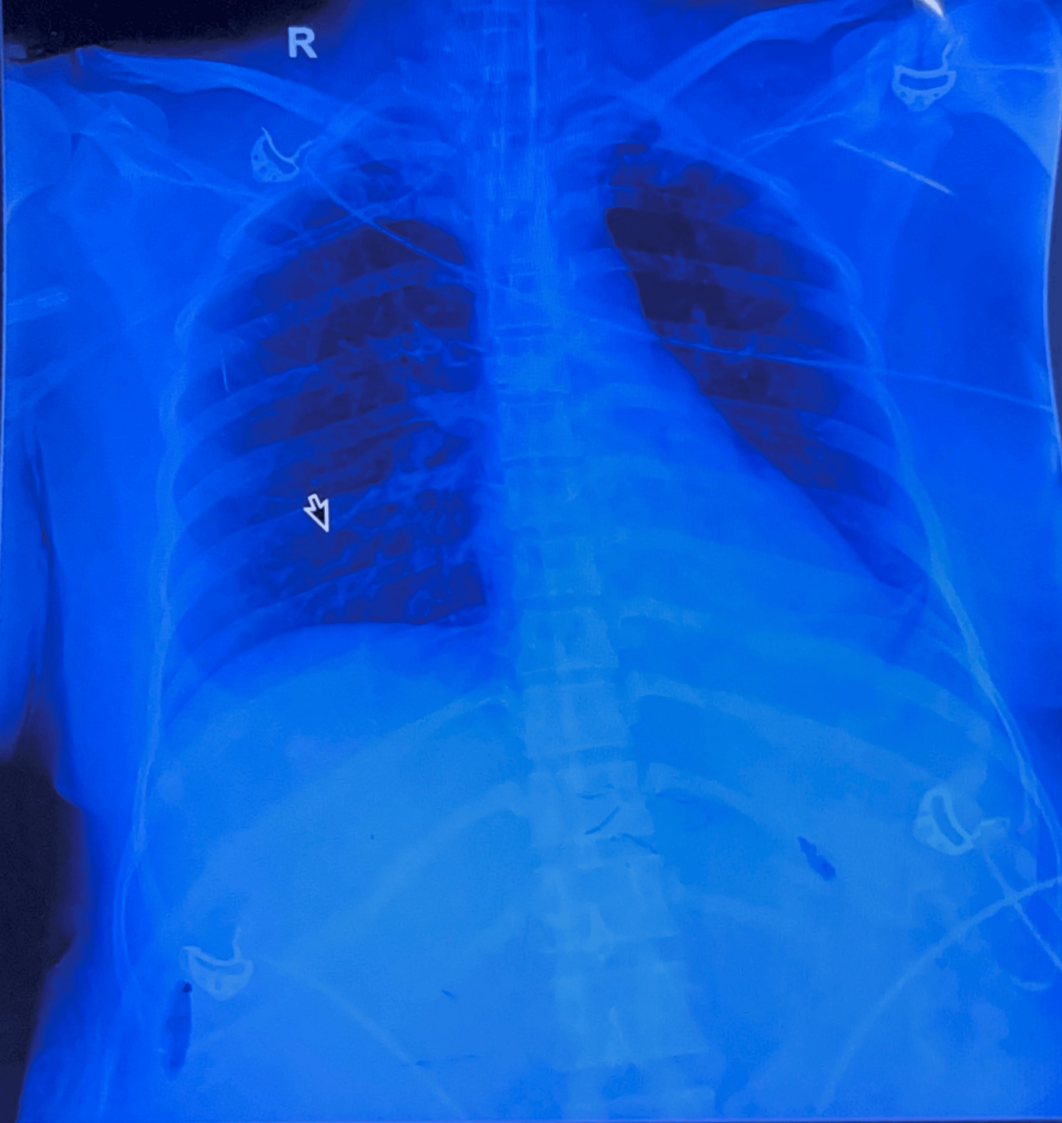
Follow-up chest X-ray taken on the fourth day of treatment showed marked resolution of the consolidative changes Arrow: marked resolution of the consolidative changes

Given the critical state and the strong clinical suspicion of GBS, treatment was initiated empirically. The patient was initiated on a five-day course of intravenous immunoglobulin (IVIG) at a dose of 0.4 g/kg/day, which was commenced within 48 hours of presentation. Concurrently, she received broad-spectrum antibiotics (piperacillin-tazobactam and azithromycin) to treat the severe CAP along with other supportive treatment. The total duration of the broad-spectrum antibiotic course was seven days. The patient initially required mechanical ventilation for four days due to respiratory muscle paralysis and respiratory distress. Then, after this acute phase, she showed clinical improvement and was successfully extubated. A follow-up chest X-ray taken on the fourth day of treatment showed marked resolution of the consolidative changes (Figure 2). She was transferred for specialized limb physiotherapy and showed remarkable recovery, achieving motor power of 4/5 in the lower limbs and 5/5 in the upper limbs by the time of discharge on hospital Day 14.

## Discussion

The definitive diagnosis of AMAN in this case was established by the characteristic electrophysiological and CSF findings. The NCV study on Day 5 of weakness confirmed a pure motor, non-length-dependent axonal loss. This pattern was evidenced by severely reduced compound muscle action potential (CMAP) amplitudes (<1 mV) in multiple motor nerves (e.g., tibial and median), while sensory nerve action potential (SNAP) amplitudes remained within the normal range. In contrast, the CSF analysis on Day 9 of weakness confirmed hallmark albuminocytologic dissociation (elevated protein, 150 mg/dL without pleocytosis). These findings fulfilled the criteria for a diagnosis of AMAN, which is a severe axonal subtype of GBS. Crucially, the combination of rapidly ascending quadriparesis, areflexia, electrophysiological confirmation, and CSF albuminocytologic dissociation establishes this case as meeting the highest diagnostic certainty, Brighton Level 1, for GBS.

However, the patient's clinical timeline differs significantly from a classical GBS pattern, presenting atypical features that complicated the initial management.

GBS is classically defined by its post-infectious interval, where neurological symptoms commence days to weeks after the initial systemic infection has cleared [5]. In this case, severe quadriparesis and respiratory failure developed simultaneously with active, radiologically confirmed CAP (on Day 4 of fever). This highly atypical "parainfectious" timing is crucial. It suggests an immediate and overwhelming immunological cross-reactivity, where the infectious agent responsible for the pneumonia and the neural tissue share common antigenic targets, potentially accelerating the immune response [6]. This rapid synchronicity bypasses the typical latency period, making the clinical picture confusing and immediately widening the differential diagnosis to include conditions like severe infectious myelitis or acute toxic neuropathies, which often present during the peak of systemic illness. The prompt progression mandated immediate initiation of IVIG without waiting for the full classical timeline to unfold.

The presence of severe, ongoing CAP and the resulting acute respiratory failure complicated the diagnosis, as critical illness polyneuropathy/myopathy (CIP/CIM) is common in the setting of prolonged sepsis and critical care. The patient's presentation prior to a lengthy ICU stay, coupled with two crucial pieces of evidence, strongly argued against CIP/CIM: normal muscle enzymes, the normal creatine phosphokinase 107.8 U/L, and creatine kinase-MB 21.9 U/L levels effectively ruled out primary myopathy or rhabdomyolysis; and the NCV pattern, the pure motor axonal pattern identified early via NCV (Day 5 of weakness), is highly specific for AMAN. While CIP can also manifest as axonal neuropathy, the rapid, ascending paralysis characteristic of GBS, in conjunction with the preserved sensory function and the eventual CSF albuminocytologic dissociation, firmly established the diagnosis of AMAN. The patient's swift motor recovery following IVIG further supported the primary immune-mediated etiology over an illness-related catabolic process.

Management hinged on a dual approach: aggressive broad-spectrum antibiotics (piperacillin-tazobactam and azithromycin) to control the severe CAP and early IVIG to address the underlying neuro-inflammatory process. The patient required mechanical ventilation for four days, highlighting the severity of respiratory involvement often seen in axonal variants, such as AMAN. This was followed by a successful extubation and impressive recovery, achieving motor power of 4/5 to 5/5 upon discharge, emphasizing the effectiveness of timely immunomodulation, even when administered with aggressive treatment for an active infection. This case highlights the importance of recognizing a treatable immune-mediated process, such as AMAN, in rapidly progressive flaccid paralysis, regardless of atypical presentation features.

Limitations

A key limitation of this case report is the lack of a comprehensive microbiological workup. Due to the acuity of the patient's critical condition and institutional constraints, specific serological tests for common GBS triggers (such as *Campylobacter jejuni*) were not performed. Consequently, the specific infectious agent that precipitated the parainfectious AMAN in this patient remains unidentified.

## Conclusions

This case report highlights a rare and potentially life-threatening presentation of AMAN where the onset of neurological deficits occurred concurrently with an acute, severe CAP. While the typical presentation of GBS involves a post-infectious interval, we must maintain a high index of suspicion for GBS and its variants, even in the setting of ongoing, severe systemic infection and atypical features and timing. Timely electrodiagnostic confirmation (AMAN pattern NCV) with biochemical support (CSF albuminocytologic dissociation) is crucial for ensuring optimal outcomes. Prompt respiratory support and early immunomodulatory treatment (IVIG) are essential components of this approach. 
